# Geriatric Fast Facts: Four Years of Growth and Reach

**DOI:** 10.1093/geroni/igaa057.703

**Published:** 2020-12-16

**Authors:** Edmund Duthie, Kathryn Denson, Deborah Simpson, Steven Denson, Michael Malone

**Affiliations:** 1 Medical College of Wisconsin, Milwaukee, Wisconsin, United States; 2 Medical College of Wisconsin, Wauwatosa, Wisconsin, United States; 3 Advocate Aurora Health, Milwaukee, Wisconsin, United States

## Abstract

Clinical teachers are increasingly challenged to find the time to provide point of care education for learners. To meet this challenge, an interprofessional team from competing health care systems created and sustained Geriatric Fast Facts (GFF): easily accessible, concise 1-2 page topic summaries for clinical teachers to use (in lieu of the mini-lecture) with learners at the point of care. Designed by geriatrics educators in consultation with IT experts, we launched a mobile enabled website that is indexed and searchable by free text or topic, organ system, ACGME competency, disease, and the “underlying science” for the disease/illness. GFF topics are authored by subject matter experts with peer review by senior geriatricians. Brief quizzes test learners’ knowledge with score reports. Our 4-year results reveal that: 1) “Geriatric Fast Facts” appear in the top Google 10 listing; 2) Search engines account for 39% of site traffic; 3) 60% of unique users (N=29,000) are via direct link; 4) 19% via referral from another site. Our “bounce rate” (55%) is ideal as users quickly gain the information sought - affirmed by our session duration which averages < 2 minutes. Our Google Analytics results reflect steady growth and international reach: 73% of sessions are from the U.S., 5% from Canada, 22% international and growing! In summary with our population continuing to age and time for teaching being limited, GFFs are quick, accessible, evidence-based resources for point of care teaching.

